# DeepPDT-Net: predicting the outcome of photodynamic therapy for chronic central serous chorioretinopathy using two-stage multimodal transfer learning

**DOI:** 10.1038/s41598-022-22984-6

**Published:** 2022-11-04

**Authors:** Tae Keun Yoo, Seo Hee Kim, Min Kim, Christopher Seungkyu Lee, Suk Ho Byeon, Sung Soo Kim, Jinyoung Yeo, Eun Young Choi

**Affiliations:** 1 Department of Ophthalmology, B&VIIT Eye Center, Seoul, South Korea; 2 Department of Ophthalmology, National Health Insurance Service Ilsan Hospital, Goyang, South Korea; 3 Department of Ophthalmology, Gangnam Severance Hospital , Institute of Vision Research, Yonsei University College of Medicine, Seoul, South Korea; 4 Department of Ophthalmology, Severance Eye Hospital, Institute of Vision Research, Yonsei University College of Medicine, Seoul, South Korea; 5 Department of Artificial Intelligence, Yonsei University, Seoul, South Korea

**Keywords:** Computational biology and bioinformatics, Diseases, Medical research

## Abstract

Central serous chorioretinopathy (CSC), characterized by serous detachment of the macular retina, can cause permanent vision loss in the chronic course. Chronic CSC is generally treated with photodynamic therapy (PDT), which is costly and quite invasive, and the results are unpredictable. In a retrospective case–control study design, we developed a two-stage deep learning model to predict 1-year outcome of PDT using initial multimodal clinical data. The training dataset included 166 eyes with chronic CSC and an additional learning dataset containing 745 healthy control eyes. A pre-trained ResNet50-based convolutional neural network was first trained with normal fundus photographs (FPs) to detect CSC and then adapted to predict CSC treatability through transfer learning. The domain-specific ResNet50 successfully predicted treatable and refractory CSC (accuracy, 83.9%). Then other multimodal clinical data were integrated with the FP deep features using XGBoost.The final combined model (DeepPDT-Net) outperformed the domain-specific ResNet50 (accuracy, 88.0%). The FP deep features had the greatest impact on DeepPDT-Net performance, followed by central foveal thickness and age. In conclusion, DeepPDT-Net could solve the PDT outcome prediction task challenging even to retinal specialists. This two-stage strategy, adopting transfer learning and concatenating multimodal data, can overcome the clinical prediction obstacles arising from insufficient datasets.

## Introduction

Central serous chorioretinopathy (CSC) is an idiopathic macular disease characterized by neurosensory retinal detachment due to serous fluid accumulation^1^. It is a rare disease with an incidence of approximately 0.21%, and it is more common among individuals in their 30 s who are socially active and under stress than among older adults^2^. Although subretinal fluid (SRF) accumulation may resolve spontaneously in some acute cases, prolonged SRF accumulation over several months can lead to serious sequelae, such as photoreceptor damage and permanent vision loss^3^. Hence, SRF absorption after treatment is the primary concern, and therapeutic strategies and follow-up intervals are based on SRF changes.

**Figure 1:**
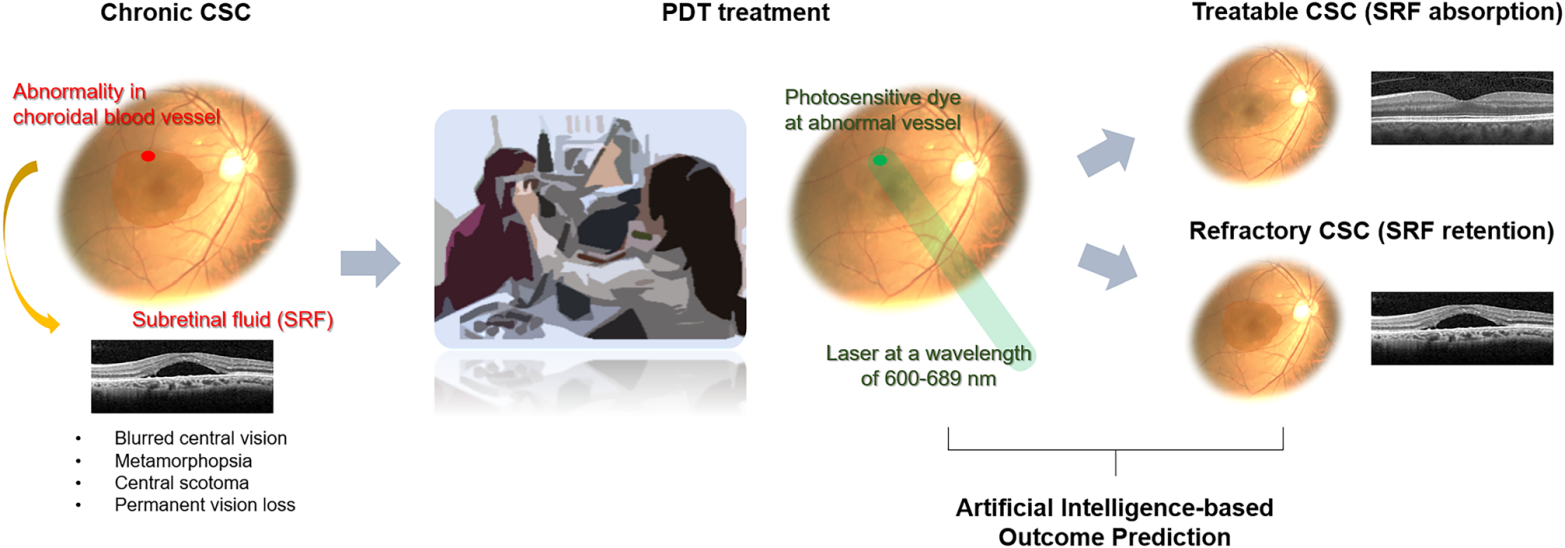
A schematic diagram of the process of photodynamic laser therapy (PDT) on central serous chorioretinopathy (CSC) and the purpose of this study.

Despite the lack of established treatment guidelines for chronic CSC^4^, photodynamic therapy (PDT) is considered safe and effective as it reduces the hyperpermeability of the choriocapillaris^5^. However, PDT is an invasive modality requiring photosensitizing agent (verteporfin) injection (Fig. 1), and the cost of the drug is exorbitant. In addition, PDT outcomes are hard to predict due to the scarce knowledge regarding clinical characteristics associated with successful PDT^6^. Moreover, different PDT regimens have not been adequately compared using large-scale systematic reviews. Therefore, devising a method for prognostic prediction is critical to decision-making regarding implementation of PDT and selection of the optimal PDT protocol.

Deep learning (DL), a type of machine learning, progressively extracts higher-level features from raw input using multiple layers. DL has shown a clinically acceptable diagnostic performance in detecting several retinal diseases, including diabetic retinopathy^7^, age-related macular degeneration^8^, and retinopathy of prematurity^9^, by extracting deep features from fundus photographs (FPs). A recent study proposed a transfer learning technique using a small amount of pathological data, which has been widely used in the medical field^10^. In the transfer learning setting, a new task with a small training dataset can be performed by considering pretrained weights from a large dataset, followed by fine-tuning the target task. In addition, various other clinical data can be integrated with the FP deep features via machine learning techniques to improve the accuracy of clinical predictions^11–13^.

CSC can be diagnosed and assessed with high accuracy using DL models based on color FPs^14^, optical coherence tomography (OCT)^15^, and en face imaging of the choroidal vasculature^16^. DL models using fundus fluorescein angiography (FA) can accurately identify the leakage points^17^, and models using OCT can distinguish chronic from acute disease^18^. Machine learning using clinical data, including features measured through multimodal imaging, exhibited high accuracy in predicting SRF absorption, indicating CSC resolution^19^. CSC can be diagnosed easily in the clinical setting by its pathognomonic manifestations of localized serous detachment of the macular neurosensory retina caused by leakage from the choroid through defects in the retinal pigment epithelium (RPE). However, predicting its post-treatment prognosis remains a challenge. To the best of our knowledge, there are no established FP-based DL prediction models for PDT outcomes due to the need for large training datasets.

Therefore, in this study, we developed a new pipeline framework based on ResNet50 and XGBoost to predict SRF absorption after PDT for CSC and identify the key impacting factors. First, we implemented ResNet50-based two-stage transfer learning for the FP domain to allow training our ResNet50 model on more domain-specific data by additionally leveraging normal FPs. Second, we developed an XGBoost model integrating the FP deep features with other clinical variables to improve the prediction accuracy. Finally, we investigated the key features affecting model prediction.

## Results

### Patients’ characteristics

Of the screened 279 eyes of 272 consecutive patients, 166 eyes of 166 patients, from Severance Eye Hospital (SEH, 147 eyes) and Gangnam Severance Hospital (GSH, 19 eyes), were included in the analysis. The patients’ mean age was approximately 52 years. The mean follow-up duration was approximately 40 months. The baseline demographic and clinical data of the treatable (n = 132) and refractory (n = 34) CSC groups are presented in Supplementary Table S1 (online). The mean age of the refractory CSC group was significantly higher than that of the treatable CSC group (56.1 vs. 51.1 years, *P* = 0.006). Other baseline characteristics did not differ significantly between the groups.

The PDT protocols implemented in the two groups were compared in Supplementary Table S2 (online). Indocyanine green angiography (ICGA)-guided PDT was more common in the treatable CSC than in the refractory CSC group (64.4% vs. 41.2%, *P* = 0.032). The proportion of patients treated with a high laser power (> 600 mW) and a high dose of verteporfin (6 mg/m^2^) was higher in the refractory CSC than in the treatable CSC group (100% vs. 88.6%, *P* = 0.039; and 29.5% vs. 11.8%, *P* = 0.035, respectively). Other PDT protocol-associated parameters did not differ significantly between the groups.

PDT outcomes are summarized in Supplementary Table S1 (“after PDT” section)**.** The central foveal thickness (CFT) was significantly lower in the treatable CSC than in the refractory CSC group (228.6 vs. 310.2 µm, *P* < 0.001). However, the distance-corrected visual acuity (DCVA) of the treatable CSC group was not significantly higher than that of the refractory CSC group. Other OCT findings and PDT-associated complications did not differ significantly between the groups.

### Transfer learning approach selection

A comparative study was performed to determine the effectiveness of stepwise transfer learning by constructing other conventional convolutional neural networks (CNNs) (Table 1). Stepwise transfer learning with ResNet50 achieved the best performance on five-fold cross-validation in the development dataset (Supplementary Fig. S1 online). The stepwise transfer learning models had higher area under the receiver operating characteristic curve (AUC) values than traditional transfer learning models, although the difference lacked statistical significance. Therefore, stepwise transfer learning with ResNet50 architecture was chosen as the CNN for our proposed model.

**Table 1:** The average performance metrics with 95% confidence intervals (CIs) for the stepwise transfer learning and other methods on the fivefold cross-validation for predicting cases of refractory central serous chorioretinopathy (CSC).

	AUC (95% CI)	Accuracy (%) (95% CI)	Sensitivity (%) (95% CI)	Specificity (%) (95% CI)	*P* value*
**Stepwise transfer learning**					
ResNet50	0.839 (0.770‒0.895)	83.0 (75.9‒88.7)	67.7 (48.6‒83.3)	87.1 (79.6‒92.6)	Ref.
VGG16	0.827 (0.756‒0.884)	83.7 (76.7‒89.3)	64.5 (45.4‒80.8)	88.8 (81.6‒93.9)	0.539
**Traditional transfer learning**					
ResNet50	0.808 (0.735‒0.868)	81.0 (73.7‒87.0)	64.5 (45.4‒80.8)	85.3 (77.6‒91.2)	0.223
VGG16	0.800 (0.726‒0.861)	80.3 (72.9‒86.4)	61.3 (42.2‒78.2)	85.3 (77.6‒91.2)	0.198
Xception	0.816 (0.744‒0.875)	81.6 (74.4‒87.5)	64.5 (45.4‒80.8)	86.2 (78.6‒91.9)	0.337
EfficientNet-b0	0.796 (0.722‒0.858)	82.3 (75.2‒88.1)	58.1 (39.1‒75.5)	88.8 (81.6‒93.9)	0.149
**Other architecture**					
NASNet-Large	0.582 (0.498‒0.663)	44.2 (36.0‒52.6)	87.1 (70.2‒96.4)	32.8 (24.3‒42.1)	0.002

*The AUCs were compared using the DeLong's method.

### DL model performance evaluation

**Figure 2:**
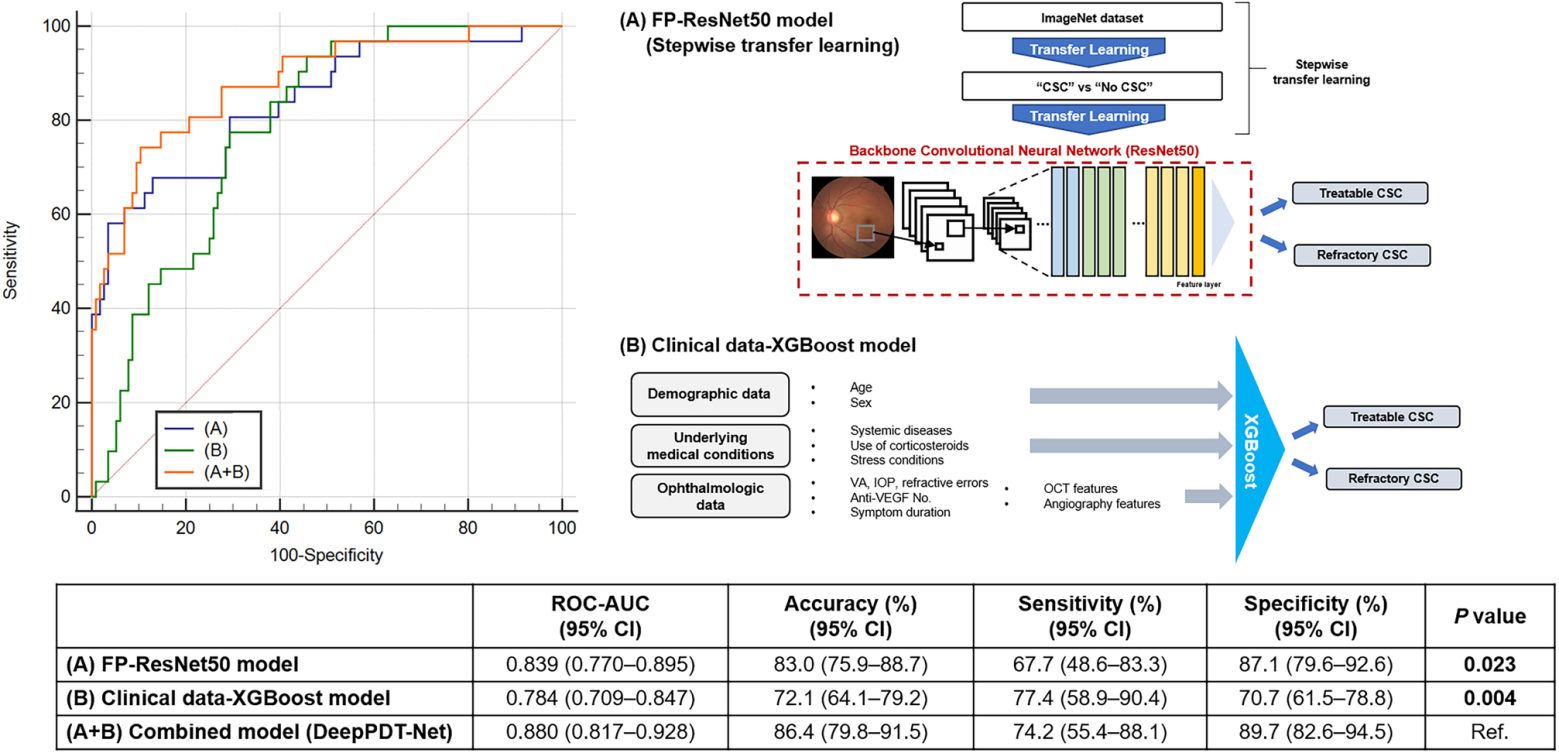
Receiver operating characteristic (ROC) curves with a table showing model performance for predicting cases of refractory central serous chorioretinopathy (CSC) in the five-fold cross validation using the Severance Eye Hospital dataset. (**A**) Domain-specific fine-tuned ResNet50 model developed using initial fundus photographs (FPs); (**B**) XGBoost model trained exclusively on clinical data; and (A + B) combined model that integrates the deep features of FPs extracted by ResNet50 and other clinical data. (A + B) DeepPDT-Net (Fig. 6) had a greater area under the curve (AUC) value of 0.880 compared with (**A**) the ResNet50 model, which had an AUC value of 0.839. CI = confidence interval. A *P* value in bold indicates statistical significance (*P* < 0.05).

The AUCs of the DL models designed using the training datasets from SEH are shown in Fig. 2. The XGBoost model trained exclusively on clinical variables showed good performance (AUC = 0.784, 95% confidence interval (CI) = 0.709‒0.847]. Interestingly, the fine-tuned ResNet50 with transfer learning on initial FPs showed excellent performance (AUC = 0.839, 95% CI = 0.770‒0.895). As expected, the concatenated model DeepPDT-Net, which horizontally connected all multimodal data, improved the performance of the algorithm (AUC = 0.880, 95% CI = 0.817‒0.928). The difference between the AUCs of the domain-specific ResNet50 and DeepPDT-Net models was significant (∆AUC = 0.0412, *P* = 0.023, DeLong's method).

External validation was performed using the test dataset from GSH to verify the generalizability of DeepPDT-Net. The model’s performance on external validation is presented in Table 2. The final combined model achieved an AUC value of 0.917 (95% CI = 0.697‒0.993).

**Table 2:** Performance of machine learning models for detecting refractory central serous chorioretinopathy cases in the external validation (Gangnam Severance Hospital dataset).

	AUC (95% CI)	Accuracy (%) (95% CI)	Sensitivity (%) (95% CI)	Specificity (%) (95% CI)	*P* value*
(A) FP-ResNet50 model	0.813 (0.570‒0.952)	68.4 (43.5‒87.4)	100.0 (29.2‒100.0)	62.5 (35.4‒84.8)	0.196
(B) Clinical data-XGBoost model	0.854 (0.619‒0.972)	79.0 (54.4‒94.0)	100.0 (29.2‒100.0)	75.0 (47.6‒92.7)	0.568
(A + B) Combined model (DeepPDT-Net)	0.917 (0.697‒0.993)	84.2 (60.4‒96.6)	100.0 (29.2‒100.0)	81.3 (54.4‒96.0)	Ref.

*AUC* Area under the receiver operating characteristic curve, *CI* Confidence interval.

*The AUCs were compared using the DeLong's method.

### Features affecting PDT outcome prediction

**Figure 3:**
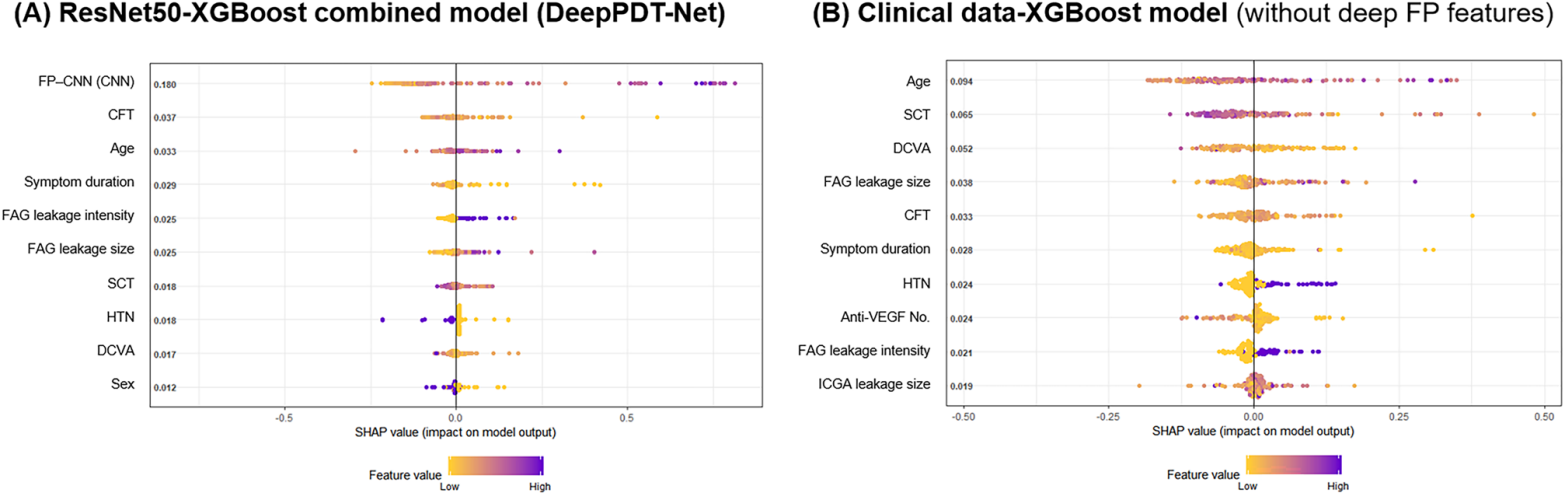
Feature importance summary plots and corresponding SHapley Additive ex-Planations (SHAP) values of (**A**) DeepPDT-Net combining ResNet50 deep features of fundus photographs (FPs) with other clinical data with XGBoost and (**B**) XGBoost model trained exclusively on clinical data without deep FP features.

The feature importance summary plots and corresponding SHapley Additive ex-Planations (SHAP) values are shown in Fig. 3. In the DeepPDT-Net model, the domain-specific FP deep features (SHAP = 0.180) were the most important, because their exclusion induced the greatest negative impact on model performance, as measured using the AUC. CFT (SHAP = 0.037) and age (SHAP = 0.033) were the second and third most important features, respectively. The intensity and size of the leakage detected using FA also had an impact on the model’s performance, but to a lesser extent. PDT protocol-associated parameters did not show any significant importance.

In the XGBoost model developed exclusively using clinical data, age (SHAP = 0.094) was the most important clinical feature affecting model performance, followed by subfoveal choroidal thickness (SHAP = 0.065) and DCVA (SHAP = 0.052).

**Figure 4:**
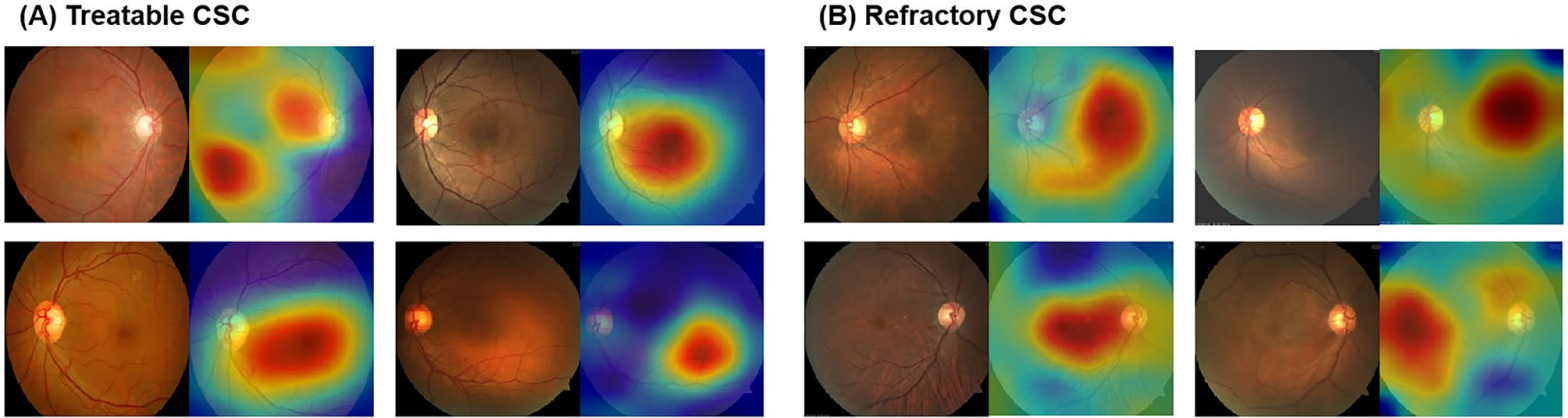
Representative gradient-weighted class activation mapping (Grad-CAM) heatmaps of fundus photography of central serous chorioretinopathy (CSC) eyes from the (**A**) treatable (complete absorption of subretinal fluid) and (**B**) refractory (retention of subretinal fluid) groups after photodynamic therapy.

The gradient-weighted class activation mapping (Grad-CAM) algorithm visualizes heatmaps of the features of interest on the FPs (Fig. 4). The color gradients of the heatmap indicated that the ResNet50-based CNN focused on the area of SRF accumulation in the input FP to predict the treatment outcomes. The CNN tended to focus on the changes in RPE lesions and subretinal deposits to detect refractory cases, rather than the SRF boundaries.

## Discussion

This study presented a novel two-stage DL pipeline strategy to predict 1-year outcomes of PDT for chronic CSC based on ResNet50 with transfer learning and horizontal concatenation with XGBoost, using initial multimodal clinical data as the input. This algorithm can be successfully used in further research to predict any outcomes of interest in a variety of ocular and systemic diseases with low prevalence and insufficient imaging data.

First, we introduced the stepwise transfer learning framework into the FP imaging domain to improve prediction performance. This resulted in high prediction accuracy of the likelihood of complete SRF absorption based on FP deep features extracted from ResNet50 with transfer learning. The transfer learning approach ensured that the pretrained ResNet50 model was more domain-specific even with the relatively smaller target CSC datasets. Therefore, two major problems of DL modeling in the biomedical imaging domain were overcome: small size of the target datasets and inequality of the classification groups^10,20^. Next, we performed horizontal concatenation of other clinical variables with the FP deep features extracted from the fine-tuned ResNet50 using XGBoost, a conventional machine learning technique^21,22^. This significantly improved the predictive accuracy of the domain-specific ResNet50. The concatenated model, DeepPDT-Net, more accurately differentiated refractory from treatable CSC. This process also revealed key features (i.e., FP deep features, CFT, and age) that primarily affected the model’s predictive ability. Finally, external validation was performed using data from another institution, which revealed that our model is robust, suggesting its potential for clinical application.

Recent studies on DL models using FPs have demonstrated satisfactory performance in reproducing the diagnosis and classification of retinal diseases frequently treated by trained clinicians in real-world situations^23^. However, previous studies have failed to sufficiently demonstrate whether FP deep features can accurately predict the prognosis of chronic or latent diseases. In this study, t-distributed stochastic neighbor embedding mapping revealed that the initial FP samples were clustered according to their classes (Supplementary Fig. S2 online). The stepwise transfer learning improved the diagnostic performance. To the best of our knowledge, the present study is the first to demonstrate not only the adequacy of the deep features extracted from a small dataset of initial FPs, but also their utmost importance for ensuring highly accurate prediction of PDT outcomes in CSC. This DL prediction based on transfer learning seems to be able to surpass the accuracy of ophthalmologists’ decision-making based on imaging interpretation. This is because it is very challenging for ophthalmologists to predict the prognosis of CSC using only initial FPs (used in the fine-tuned ResNet50 with transfer learning) as these provide relatively limited information to predict treatment outcomes.

Our transfer learning-based approach was effective for training on the small CSC FP dataset. Transfer learning has been demonstrated to be a robust and efficient technique in a wide range of dataset sizes, ranging from large datasets with 10,000 images^24^ to small datasets with approximately 10 training samples^25^. Several previous studies have shown that transfer learning is better than conventional training protocols with fully-trained weight (with scratch) for small FP datasets^26,27^. Transfer learning can provide more sensitive and specific performance on small datasets (< 10,000 training images) because conventional training easily faces overfitting or convergence problems during training^28^. It should be noted that training on very large datasets (> 100,000 images) using conventional training with scratch generally shows better than transfer learning as more image patterns can be detected using the whole network parameters.

FP is a cost-effective and non-invasive procedure that can easily depict the details of the retina in clinical settings. Therefore, ResNet50, which was fine-tuned using the initial FPs, possesses simplicity and sufficient utility to facilitate clinical application without the need for additional examination or analysis. The use of this prediction model for patients with CSC will enable clinicians to make evidence-based decisions and provide accurate explanations when contemplating PDT treatment. According to our results, pre-PDT baseline parameters, such as FP deep features, CFT, and age, are more critical to the model for predicting treatment outcomes than PDT protocol-associated parameters.

This study has some limitations that can be overcome by future research. First, the DL model trained and developed in this study focused only on predicting SRF absorption. Further studies are required to develop additional DL models that can predict other clinically significant prognoses, such as the final visual acuity, time to complete SRF absorption, and SRF recurrence. Second, it is important to elucidate the mechanism of action of DL using existing and future methodologies to improve the acceptability of the DL system in the clinical environment. In this study, Grad-CAM heatmaps were generated to highlight the regions of influence on FPs, which contributed to the algorithm conclusion. We assumed that the ResNet50-based CNN detected CSC by assessing the location, area, and gradient of SRF accumulation on FPs. However, ResNet50 fined-tuned with transfer learning seemed to be more focused on subretinal deposits and RPE changes, particularly for the prediction of refractory disease. Nevertheless, the map is difficult to interpret and is limited in its “explainability” to ophthalmologists. Third, although various clinical parameters (age, CSC duration, initial vision, choroid features on OCT, and verteporfin dose) have been proposed as prognostic factors, there is no consensus about how clinicians can predict PDT outcome based on these. Moreover, as mentioned above, FPs provide little information to predict the outcome of PDT, even for retina specialists. Therefore, the strength of agreement regarding the prediction task between the DeepPDT-Net and clinicians could not be analyzed in this study.

In conclusion, by adopting the stepwise transfer learning strategy to the pretrained ResNet50, DeepPDT-Net could predict the success or failure of PDT for chronic CSC with significantly high accuracy, even with a relatively small dataset of FPs. Moreover, XGBoost, which concatenated FP deep features with other clinical data, not only improved the prediction accuracy but also elucidated the degree of the effect of the key factors on model performance. DeepPDT-Net may allow clinicians to make more rational treatment decisions by considering the cost-effectiveness of PDT in patients with CSC. Additionally, the treatment prognosis could be explained to patients in advance, thereby increasing treatment compliance. This two-stage strategy based on the stepwise ResNet50 transfer learning algorithm to extract deep features from imaging results and horizontal concatenation of clinical variables using XGBoost can be applied relatively easily to various clinical research fields.

## Methods

### Study design and population

This study was approved by the Institutional Review Board of Severance Hospital (approval no. 4-2020-1357) and conformed to the tenets of the Declaration of Helsinki. Informed consent for PDT was obtained from all patients. The requirement for informed consent for the present study was waived owing to its retrospective nature.

We retrospectively reviewed the medical records and multimodal images of consecutive patients who underwent PDT for chronic or recurrent CSC between November 2008 and December 2020 at SEH and at the Ophthalmology Department of GSH. Chronic or recurrent CSC was defined as the presence of SRF for at least 6 months, focal leakage or RPE changes observed on FA, and abnormally dilated choroidal vessels on ICGA. The exclusion criteria were as follows: (1) presence of any other retinal disease, including age-related macular degeneration, pathologic myopia, retinal vascular disorder, diabetic retinopathy, and hypertensive retinopathy; (2) presence of media opacity, including cataract and vitreous or corneal opacity; (3) other treatment for CSC, such as focal laser photocoagulation or anti-vascular endothelial growth factor injection, within 3 months of PDT; and (4) unavailable 12-month follow-up ophthalmologic data. Patients with missing data (history of treatment or FPs) or low-quality FPs were also excluded. If both eyes met the eligibility criteria, only the right eye was included in the analysis.

Additionally, we used 745 FPs from healthy control eyes for the stepwise transfer learning process. The ophthalmologic data from SEH (147 eyes for the target task) and healthy eyes (745 eyes for an additional learning dataset) comprised the dataset for model construction and calibration, i.e., the training dataset. In the training dataset, we used five-fold cross-validation, which is the currently preferred technique in the field of machine learning to assess objective performance without selection bias. The data from GSH (19 eyes) comprised the external dataset for independent validation of the developed DL model.

### Data collection

We extracted and recorded the patients’ demographic and basic clinical characteristics, including age, sex, underlying medical conditions (e.g., hypertension, hyperlipidemia, and diabetic mellitus), use of corticosteroids, stress conditions (e.g., sleep disorder and shift work), and other relevant ocular history details, such as CSC duration and prior treatments.

Ophthalmologic data were obtained from measurements of DCVA, refractive errors, and intraocular pressure; FPs; spectral-domain OCT (Spectralis; Heidelberg Engineering, Heidelberg, Germany); and FA and ICGA (Heidelberg Retinal Angiograph II, Heidelberg Engineering) performed before PDT (baseline), 1‒3 and 11‒13 months after PDT, and at the last visit. DCVA was measured using the Snellen chart and converted to the logarithm of the minimal angle of resolution for analysis. CFT was measured automatically using the built-in software in the OCT device. Subfoveal choroidal thickness was assessed as the distance from Bruch’s membrane to the choroid–sclera junction on enhanced depth images. The presence of RPE detachment was recorded. The size (µm) and intensity (low, as fluorescent as the perifoveal vessels; and high, more fluorescent than the perifoveal vessels) of focal leakage or hyperfluorescent areas on mid-phase FA and ICGA were measured.

The following PDT protocol parameters were recorded: (1) guidance angiography (FA or ICGA), (2) location of the targeted lesion (subfoveal, perifoveal, or extrafoveal), (3) spot size (≥ or < 4,500 µm), (4) exposure time (42 or 83 s), (5) laser power (≥ or < 600 mW/cm^2^), (6) fluence rate (12, 25, or 50 J/cm^2^), and (7) dose of verteporfin (Visudyne®; Novartis AG, Basel, Switzerland) (2, 3, or 6 mg/m^2^). Possible PDT-associated complications were also documented, including secondary choroidal neovascularization, chorioretinal atrophy, choroidal infarction, and optic atrophy.

PDT efficacy was evaluated primarily based on the complete SRF absorption on OCT, and secondarily, based on the DCVA changes at the 12-month follow-up. SRF absorption on OCT after PDT was designated as treatable disease, whereas SRF retention after treatment was considered to indicate refractory disease.

### Machine learning algorithms

**Figure 5:**
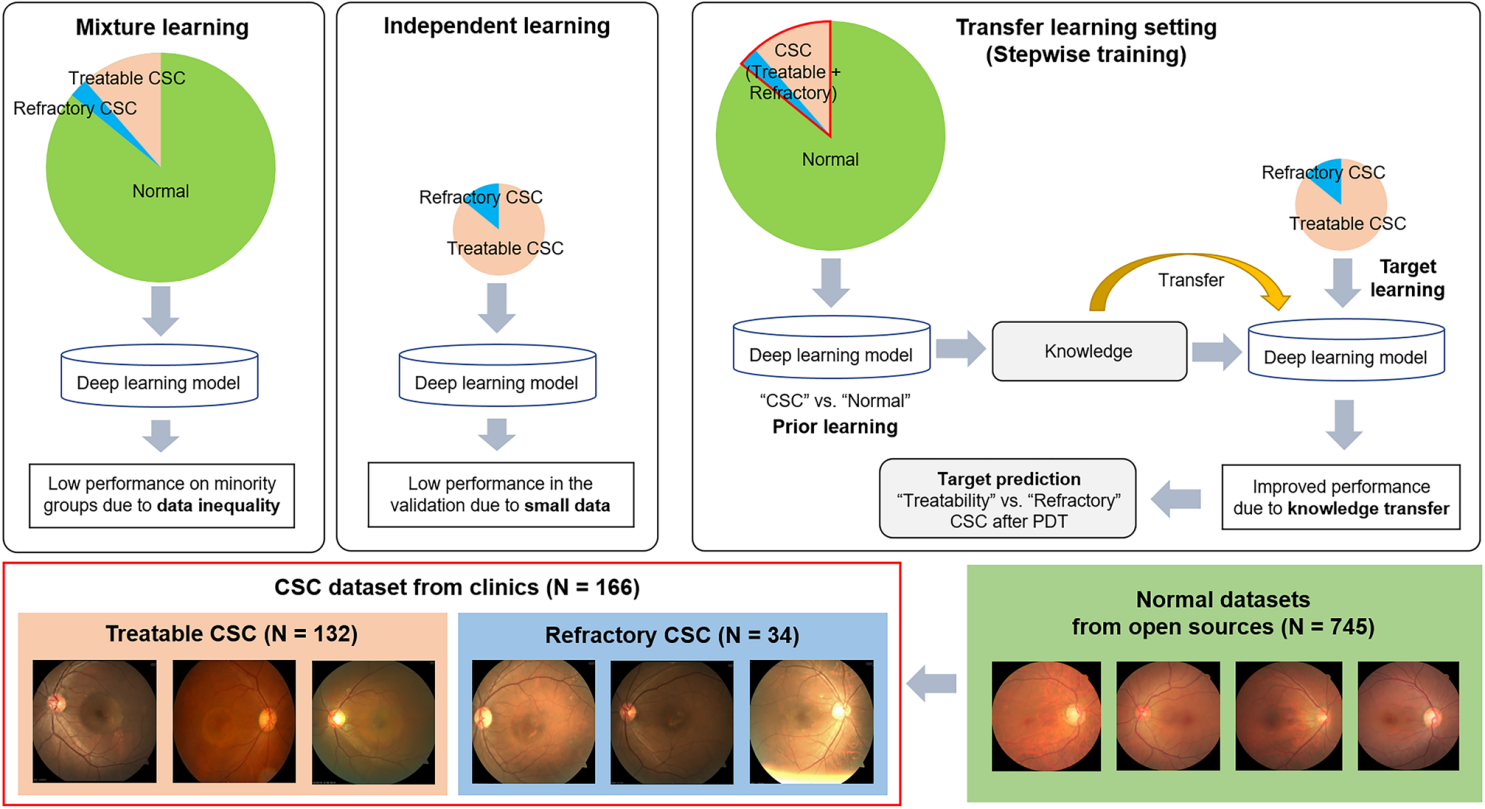
The stepwise transfer learning scheme to classify treatable and refractory central serous chorioretinopathy (CSC) for photodynamic laser therapy (PDT).

We employed a stepwise transfer learning framework to predict PDT outcomes to overcome the lack of sufficient clinical data. Namely, as CSC is a relatively rare disease (Supplementary Table S3 online), PDT is performed sporadically in ophthalmology clinics. Furthermore, direct transfer learning from ImageNet to a target containing a very small amount of data can cause overfitting or non-convergence of training. The stepwise transfer learning technique has been used in the lung computed tomography^29^ and musculoskeletal X-ray^30^ imaging domains to improve diagnostic performance by incorporating ImageNet and prior domain knowledge into their tasks (Supplementary Fig. S3 online). Since the original CSC dataset was too small to solitarily train the CNN, we adopted the domain adaption approach based on stepwise transfer learning to deal with data inequality or insufficiency^10^. In the first stage (prior learning), we trained the model to distinguish CSC (all cases, treatable and refractory) from normal FPs using the whole dataset via transfer learning using a pretrained CNN from the ImageNet dataset. In the second stage (target learning), the model was fine-tuned using only the treatable and refractory CSC datasets. The objectives of stepwise transfer learning and the algorithm development processes are illustrated in Fig. 5.

We used a DL approach with the stepwise transfer learning framework to build a classification model to identify eyes with refractory CSC. The DL model consisted of two parts: a CNN feature encoder and a concatenating XGBoost model, similar to that used in a previous study^31^. A pretrained CNN based on ResNet50 architectures was adopted as the feature extractor. Initially, this CNN architecture was pretrained on the ImageNet database and imported into the MATLAB R2020b platform (MathWorks Inc., Natick, MA, U.S.A.) (Supplementary Table S4 online). The input images were resized to the original input tensor of the ResNet50 architecture (224 × 224 pixels). In the first stage of prior learning, after the ResNet50 layers and weights were unfixed on ImageNet, we trained a binary classification model by fine-tuning the ResNet50 weights. The goal of this training was to temporarily determine the presence or absence of CSC on FPs for domain adaption^20^ (training data including FPs of 147 eyes with CSC from SEH vs. external data including 745 healthy control FPs)^32,33^.

The ResNet50 weights of the feature extractor were fixed after the initial training during the second stage, i.e., target learning. In our task, the last layers of the CNN architecture were replaced by a modified fully connected layer (with 2 × 2048 weights and 2 × 1 bias for ResNet50) and a SoftMax function layer for binary classes, which set the output of the classification result to a range of 0–1, corresponding to the predicted probability of treatable and refractory CSC. Additional fine-tuning of the fully connected layer and the SoftMax layer for the final task was performed using the refractory CSC (31 eyes from SEH) and treatable CSC (116 eyes from SEH) datasets. Therefore, the initial training task (determination of the presence or absence of CSC) was considered as the source domain knowledge to train the final model via transfer learning. The target images were projected through the transferred projection function of the bridge dataset (CSC vs. healthy FPs). The output data of the trained ResNet50 with domain adaption were concatenated before serving as input data for the XGBoost model, akin to the approach used in a previous study^21^.

Data augmentation was performed using linear transformation, including left and right flips, width and height translation from − 30 to + 30 pixels, random rotation from − 30 to 30°, zooming from − 15 to 20%, and random image shearing change from − 15 to 15° to prevent overfitting during training. The model was optimized using the cross-entropy loss function and stochastic gradient descent setting with a batch size of 20 and learning rate of 0.0001. Maximum epochs were set at 25 during prior learning (the first stage of transfer learning) and 50 during target learning (the second stage of transfer learning). The cross-entropy loss function for the CNN classification task was defined as follows:

$$L_{Cross Entropy} = - \mathop \sum \limits_{i}^{N} p_{i} {\text{log}}\left( {q_{i} } \right)$$

where ${p}_{i}$ represents the ground truth value (CSC or healthy eyes in the first stage, and treatable or refractory CSC in the second stage), and ${q}_{i}$ represents the predicted probability value from a classifier for the $i$ th image.

**Figure 6:**
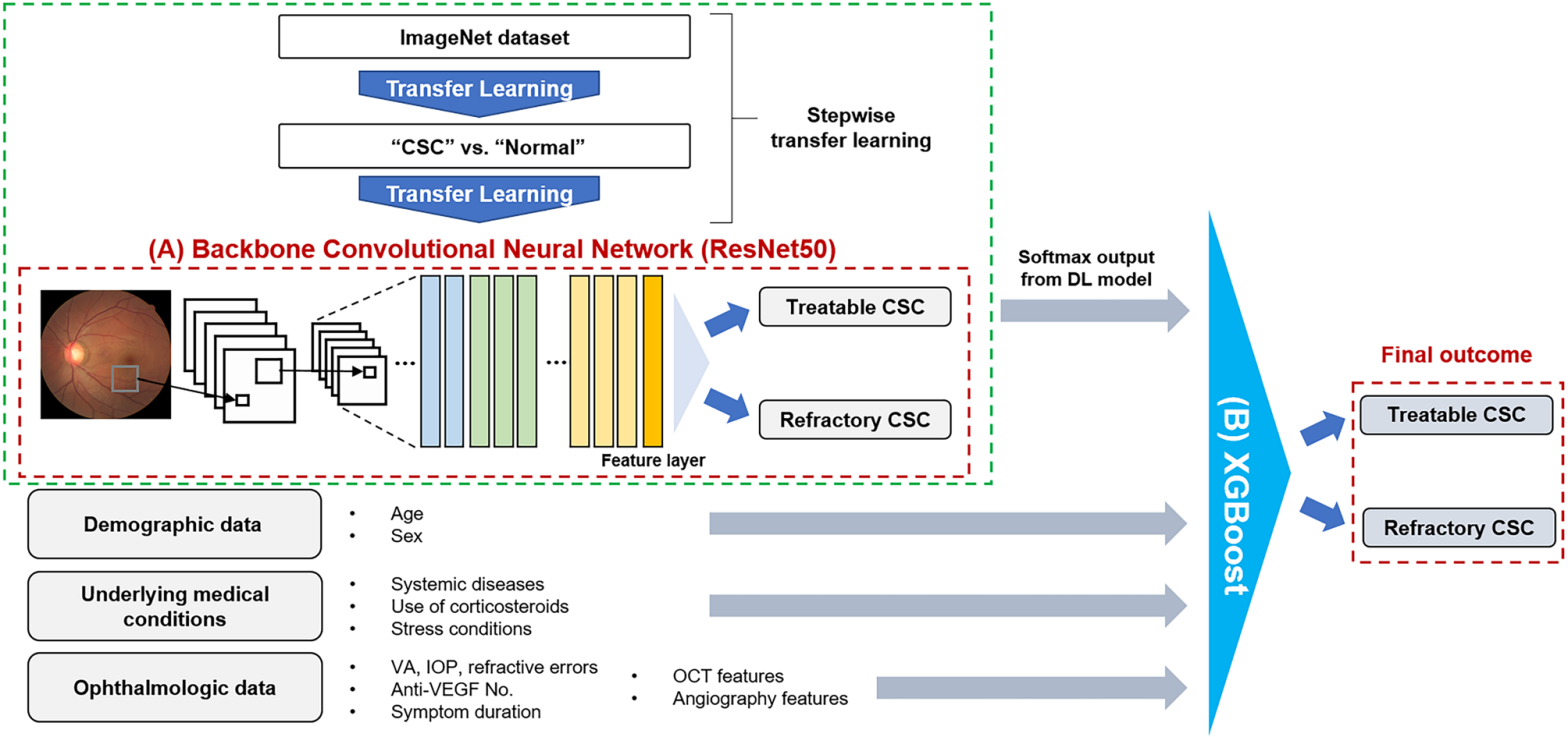
Schematic diagram explaining the structure of DeepPDT-Net: the backbone ResNet50 model for deep transfer learning from initial fundus photographs (FPs) and the XGBoost model for concatenation of the extracted deep FP features with other multimodal clinical data.

We built DeepPDT-Net to combine all predictions across the CNN features gleaned from the stepwise transfer learning and clinical variables. We employed the XGBoost approach to horizontally concatenate multimodal clinical data (Fig. 6). XGBoost was employed to detect refractory CSC using FPs and clinical features to analyze the final outcome of this study. XGBoost training entailed the use of a fine-tuning algorithm for the specific task of detecting refractory CSC. The SHAP technique was adopted to explain the output of the XGBoost model^20^. The SHAP technique calculates the contribution of each input variable in each decision of a given machine learning model.

To evaluate the performance of the developed DL models (domain-specific FP–ResNet50, clinical data–XGBoost, and combined DeepPDT-Net), we calculated the AUCs and compared them using the DeLong's method provided in MedCalc 20 (MedCalc, Mariakerke, Belgium). Youden’s index, which is a widely used estimate of the optimal threshold that gives equal weight to sensitivity and specificity, was adopted to set a cutoff value for responsive or refractory CSC results to PDT. All statistical tests were two-sided. Statistical significance was set at *P* < 0.05.

## Supplementary Information

Supplementary Information.

## Data Availability

Prof. EY. C. had full access to all the data in the study and takes responsibility for the integrity of the data and the accuracy of the data analysis. The patients’ data will not be available to the public due to privacy concerns. The code used to generate the results are available by contacting the corresponding author upon reasonable request.
