# Supplementary material for: DeepPDT-Net: predicting the outcome of photodynamic therapy for chronic central serous chorioretinopathy using two-stage multimodal transfer learning
Source: Sci Rep. 2022 Nov 4;12:18689. doi: 10.1038/s41598-022-22984-6 (PMC9636239; doi:10.1038/s41598-022-22984-6)
Supplement: Supplementary file 1 — Supplementary Information. [file 41598_2022_22984_MOESM1_ESM.docx]

**DeepPDT-Net: Predicting the Outcome of Photodynamic Therapy for Chronic Central Serous Chorioretinopathy Using Deep Transfer Learning**

**SUPPLEMENTARY FIGURES**


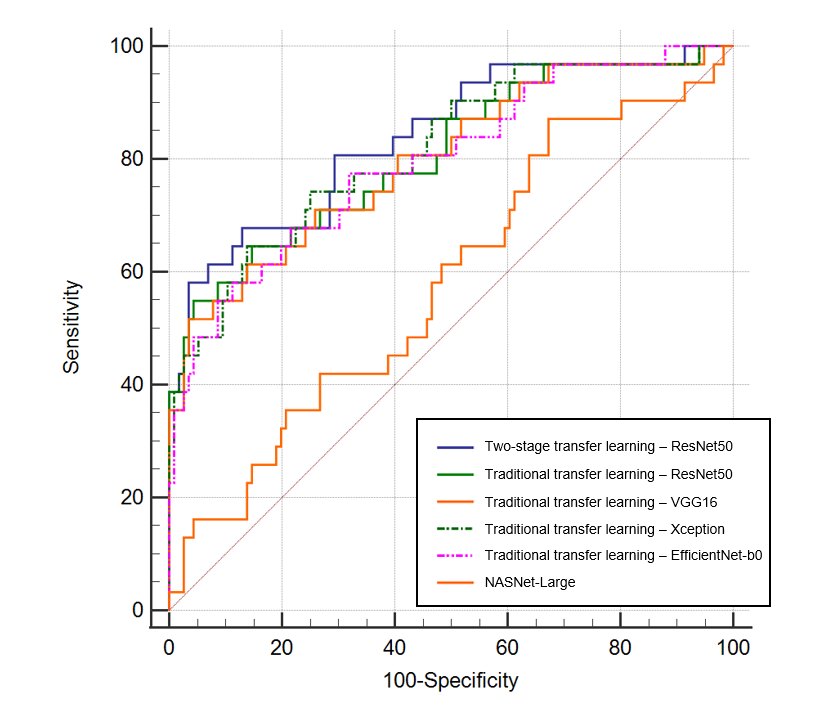


**Supplementary Figure S1.** Receiver operating characteristic curves for the two-stage transfer learning and other methods on the 5-fold cross-validation for predicting cases of refractory central serous chorioretinopathy (CSC)


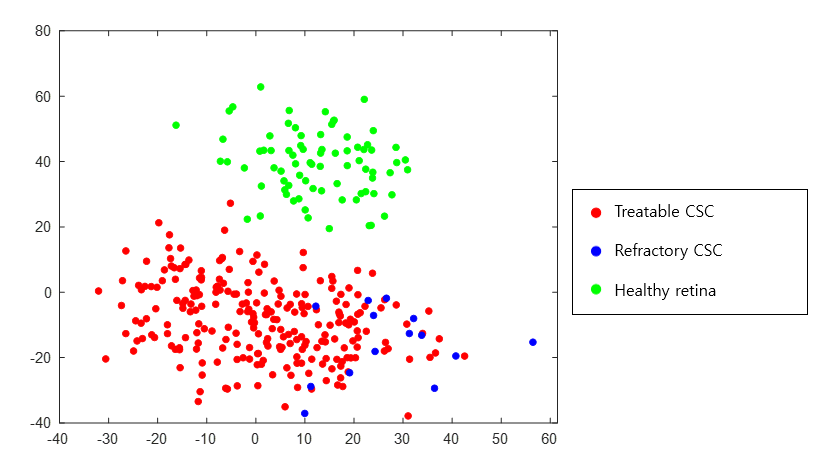


**Supplementary Figure S2.** The feature space visualized using the 2D t-distributed stochastic neighbor embedding technique.

The feature space was generated using the ResNet-50 pretrained to ImageNet.


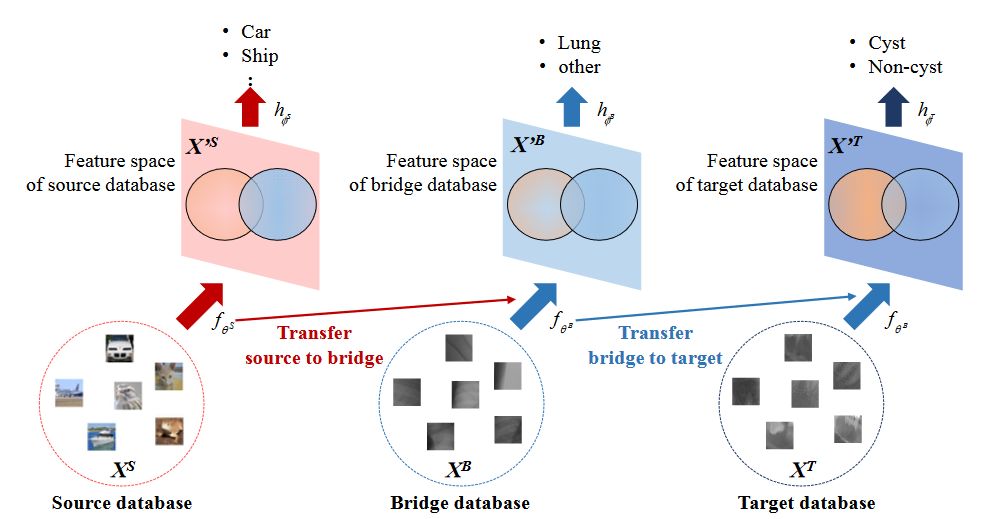


**Supplementary Figure S3.** Schematic of the concept and processes involved in two-stage deep transfer learning for medical image analysis

The source of the picture is as follows: “Modality-bridge Transfer Learning for Medical Image Classification”, CISP-BMEI 2017 (<https://arxiv.org/ftp/arxiv/papers/1708/1708.03111.pdf>).

**SUPPLEMENTARY TABLES**

**Supplementary Table S1.** Comparison of demographics and clinical characteristics of the treatable and refractory central serous chorioretinopathy (CSC) after photodynamic therapy (PDT).

|  | **Treatable CSC**  **(N = 132)** | **Refractory CSC**  **(N = 34)** | ***P* value** |
| --- | --- | --- | --- |
| **Age (years)** | 51.1±9.4 | 56.1±9.9 | **0.006** |
| **Gender, male** | 85 (64.4) | 19 (55.9) | 0.360 |
| **Laterality, right** | 55 (41.7) | 10 (29.4) | 0.192 |
| **CSC duration (months)** | 24.4 ± 27.4 | 21.5 ± 23.9 | 0.573 |
| **Follow-up duration (months)** | 41.9 ± 37.9 | 38.2 ± 36.2 | 0.607 |
| **Before PDT** | | | |
| **Distance corrected visual acuity (LogMAR)** | 0.3 ± 0.3 | 0.2 ± 0.2 | 0.204 |
| **Optical coherence tomography findings** |  |  |  |
| **Pigment epithelial detachment, yes** | 26 (19.7) | 7 (20.6) | 0.874 |
| **Central foveal thickness (micrometer)** | 408.1 ± 125.8 | 389.8 ± 93.6 | 0.437 |
| **Subfoveal choroidal thickness (micrometer)** | 410.7 ± 114.3 | 372.3 ± 125.6 | 0.096 |
| **Fluorescein angiography fidings** |  |  |  |
| **Focal leakage, yes** | 122 (92.4) | 32 (94.1) | 0.225 |
| **Size of hyperfluorescence (micrometer)** | 1728.5 ± 1267.7 | 2319.3 ± 1568.8 | 0.027 |
| **High-intensity, yes** | 30 (22.7) | 13 (38.2) | 0.079 |
| **Indocyanine green angiography findings** |  |  |  |
| **Hyperfluorescence, yes** | 107 (81.1) | 25 (73.5) | 0.383 |
| **Size (micrometer)** | 2430.6 ± 1180.3 | 2745.1 ± 1303 | 0.213 |
| **High-intensity, yes** | 22 (16.7) | 6 (16.9) | 0.547 |
| **After PDT** | | | |
| **Distance corrected visual acuity (LogMAR)** | 0.2 ± 0.3 | 0.3 ± 0.3 | 0.124 |
| **Optical coherence tomography findings** |  |  |  |
| **Pigment epithelial detachment, yes** | 15 (11.4) | 9 (26.5) | 0.075 |
| **Central foveal thickness (micrometer)** | 228.6 ± 41.4 | 310.2 ± 97.5 | **<0.001** |
| **Subfoveal choroidal thickness (micrometer)** | 344.7 ± 123.6 | 330.4 ± 127.5 | 0.553 |
| **Complication associated with PDT** |  |  |  |
| **Chorioretinal atrophy, yes** | 5 (3.8) | 1 (2.9) | 0.814 |
| **Choroidal neovascularization, yes** | 16 (12.1) | 5 (14.7) | 0.686 |

All data listed as mean ± standard deviation or number of cases (%).

Chi-square test was used for the categoric variables and independent t-test was used for the continuous variables. A P-value in bold indicates statistical significance (p < 0.05).

**Supplementary Table S2.** Comparison of photodynamic therapy (PDT) protocols between treatable and refractory central serous chorioretinopathy (CSC) after treatment

|  | **Treatable CSC**  **(N = 132)** | **Refractory CSC**  **(N = 34)** | ***P* value** |
| --- | --- | --- | --- |
| **Guided angiography** |  |  | **0.032** |
| **Fluorescein angiography** | 43 (32.6) | 17 (50.0) |  |
| **Indocyanine green angiography** | 85 (64.4) | 14 (41.2) |  |
| **Location of hyperfluorescence’s center** |  |  | 0.288 |
| **Subfovea** | 95 (72.0) | 21 (61.8) |  |
| **Perifovea** | 31 (23.5) | 9 (26.5) |  |
| **Extrafovea** | 3 (2.3) | 1 (2.9) |  |
| **Spot size (micrometer)** | 3673.2 ± 1710 | 4109.7 ± 1874.3 | 0.215 |
| **Laser exposure time (seconds)** |  |  | 0.081 |
| **42** | 18 (13.6) | 1 (2.9) |  |
| **83** | 114 (86.4) | 33 (97.1) |  |
| **Laser power (mW/cm^2^)** |  |  | **0.039** |
| **< 600** | 15 (11.4) | 0 (0) |  |
| **≥ 600** | 117 (88.6) | 34 (100) |  |
| **Fluence (J/cm^2^)** |  |  | 0.592 |
| **25** | 33 (25.0) | 7 (20.6) |  |
| **50** | 99 (75.0) | 27 (79.4) |  |
| **Visudyne dose (mg/m^2^)** |  |  | **0.035** |
| **3** | 93 (70.5) | 30 (88.2) |  |
| **6** | 39 (29.5) | 4 (11.8) |  |

All data listed as mean (standard deviation) or frequency (%).

Chi-square test was used for the categoric variables and independent t-test was used for the continuous variables. A P-value in bold indicates statistical significance (p < 0.05).

**Supplementary Table S3.** Characteristics and prevalence of central serous chorioretinopathy (CSC) in the present study

| Disease | Imaging Characteristics^1^ | Prevalence (per 10,000 individuals) | Rare disease category |
| --- | --- | --- | --- |
| CSC | OCT: Subretinal fluid under the central macula, foveal distortion  Fluorescein angiography: An expanding point of fluorescein leakage under an area of serous detachment of the neurosensory retina, without signs of subretinal neovascularization  Indocyanine green angiography: Segments with late choroidal hyperpermeability also showing a delay in filling. | 0.061% ^2^ | Definitely a rare disease* |

* Included in the Orphanet rare disease database.^3^

**References**

1. Wang M, Munch IC, Hasler PW, Prunte C, Larsen M. Central serous chorioretinopathy. *Acta Ophthalmol.* 2008;86(2):126-145.

2. Rim TH, Kim HS, Kwak J, Lee JS, Kim DW, Kim SS. Association of Corticosteroid Use With Incidence of Central Serous Chorioretinopathy in South Korea. *JAMA Ophthalmol.* 2018;136(10):1164-1169.

3. Nguengang Wakap S, Lambert DM, Olry A, et al. Estimating cumulative point prevalence of rare diseases: analysis of the Orphanet database. *Eur J Hum Genet.* 2020;28(2):165-173.

**Supplementary Table S4.** The backbone code for training ResNet-50 (task learning)

The code was properly modified for adopting transfer learning processes and the corresponding datasets in each step.

| imds = imageDatastore(path,true);  tbl = countEachLabel(imds)  [imdsTrain,imdsValidation, imdsTest] = splitEachLabel(imds, 0.7, 0.1, 'randomize');    % net = resnet50;  load net; %load the network pretrained in the prior learning  inputSize = net.Layers(1).InputSize;  lgraph = layerGraph(net);    numClasses = numel(categories(imdsTrain.Labels));    newfclayer = fullyConnectedLayer(numClasses,...  'Name', 'new_fc',...  'WeightLearnRateFactor',10,...  'BiasLearnRateFactor',10);  lgraph = replaceLayer(lgraph, net.Layers(end-2).Name, newfclayer);    newclasslayer = classificationLayer('Name', 'new_classoutput');  lgraph = replaceLayer(lgraph, net.Layers(end).Name, newclasslayer);    analyzeNetwork(net)  pixelRange = [-30 30];  scaleRange = [0.85 1.2];  imageAugmenter = imageDataAugmenter( ...  'RandRotation',[-30 30],...  'RandXReflection',true, ...  'RandXShear',[-15,15],...  'RandYShear',[-15,15],...  'RandXTranslation',pixelRange, ...  'RandYTranslation',pixelRange, ...  'RandXScale',scaleRange, ...  'RandYScale',scaleRange);  augimdsTrain = augmentedImageDatastore(inputSize(1:2),imdsTrain, ...  'DataAugmentation',imageAugmenter, 'ColorPreprocessing', 'gray2rgb');    augimdsValidation = augmentedImageDatastore(inputSize(1:2),imdsValidation, 'ColorPreprocessing', 'gray2rgb');    miniBatchSize = 20;  valFrequency = floor(numel(augimdsTrain.Files)/miniBatchSize);  options = trainingOptions('sgdm', ...  'MiniBatchSize',miniBatchSize, ...  'MaxEpochs',50, ...  'InitialLearnRate',1e-4, ...  'Shuffle','every-epoch', ...  'ValidationData',augimdsValidation, ...  'ValidationFrequency',valFrequency, ...  'Verbose',false, ...  'Plots','training-progress');    net = trainNetwork(augimdsTrain,lgraph,options);    augimdsTest = augmentedImageDatastore(inputSize(1:2), imdsTest, 'ColorPreprocessing', 'gray2rgb');  [predictedClasses, predictedScores] = classify(net, augimdsTest);  accuracy = mean(predictedClasses == imdsTest.Labels) |
| --- |
